# Training in focused echocardiography for intensive care specialists: can delivery meet perceived need?

**DOI:** 10.1186/cc9449

**Published:** 2011-03-11

**Authors:** G McNeill, A Whiteside, A Tridente, D Bryden

**Affiliations:** 1 Sheffield Teaching Hospitals, Sheffield, UK; 2 Sheffield Teaching Hospitals NHS Trust, Sheffield, UK

## Introduction

There is increasing recognition of the utility of focused echocardiography in critically ill patients and a need for suitable training programmes to be developed to meet the specific needs of critical care. Critical care communities across Europe have struggled to implement focused echocardiography into everyday clinical practice. We aim to determine whether a training programme could be implemented during a year of advanced intensive care training in a region where none of the critical care consultant body had accreditation in echocardiography, and to establish the perceived training requirements in critical care echocardiography in our region and to evaluate what information clinicians wished to obtain from a focused echocardiography examination.

## Methods

Trainees attended a course designed for echocardiography in a peri-arrest situation. Local cardiac anaesthetists with experience in transthoracic echocardiography were recruited as mentors. Data archiving protocols were established. Trainees performed an initial 10 scans directly supervised on the cardiac ICU. A further 40 scans were completed independently on the general ICU. A logbook was maintained and the scans reviewed with a mentor prior to final sign off. This process was supported by a regional educational meeting where personnel interested in echocardiography reviewed the types of training provided and how this matched local needs and resources. This included trainees and trainers in intensive care medicine, anaesthesia and acute medicine.

## Results

Although 91% of doctors wished to incorporate focused echocardiography into their clinical practice, only 36% had undergone any focused echocardiography training and only 5% had focused echocardiography accreditation. The majority of respondents wished only to incorporate eyeball assessments of ventricular function but did not wish to perform more complex examinations such as Doppler assessment.

## Conclusions

It is possible to implement a simple training programme in echocardiography in an intensive care medicine department with no prior experience in critical care echocardiography. Within our region there is strong demand for simple training in focused echocardiography rather than a higher level of accreditation currently offered by many courses.
